# Prognostic alternative splicing regulatory network of RBM25 in hepatocellular carcinoma

**DOI:** 10.1080/21655979.2021.1908812

**Published:** 2021-04-08

**Authors:** Yong-Fa Zhang, Yi-Xiu Wang, Ning- Zhang, Zhen-Hai Lin, Long-Rong Wang, Yun Feng, Qi Pan, Lu Wang

**Affiliations:** a Department of Hepatic Surgery, Fudan University Shanghai Cancer Center, Shanghai, China; b Department of Oncology, Shanghai Medical College, Fudan University, Shanghai, China

**Keywords:** hepatocellular carcinoma, RBM25, overall survival, regulatory network, prognosis

## Abstract

RNA-binding motif protein 25 (RBM25) is a poorly characterized RNA-binding protein that is involved in several biological processes and regulates the proliferation and metastasis of tumor cells. The regulatory role of RBM25 in hepatocellular carcinoma (HCC) is unknown. Here, RBM25 expression and outcomes in HCC patients were evaluated using The Cancer Genome Atlas database. RBM25 was overexpressed in HCC patients compared with the healthy group. The high expression of RBM25 in tumor tissues was significantly related to poor overall survival (P<0.001). Overexpression of RBM25 significantly contributed to poorer survival in male patients and N0 stage patients (P<0.001). Spearman analysis and weighted gene co-expression network analysis identified 694 RBM25-related genes. Protein-protein interaction network analysis revealed the Cluster with the highest score, which positively correlated with RBM25. CDCA5 and INCENP were identified as the core functional genes related to RBM25. The overexpression of CDCA5 and INCENP in HCC patients was examined using the Human Protein Atlas database. The findings collectively indicated that RBM25 may interact with CDCA5 and INCENP to regulate HCC. Our detailed characterization of RBM25 protein interactions and related core functional genes provides a basis for further studies aimed at identifying molecular regulatory pathways or splicing events.

## Graphical abstract

**Figure:**
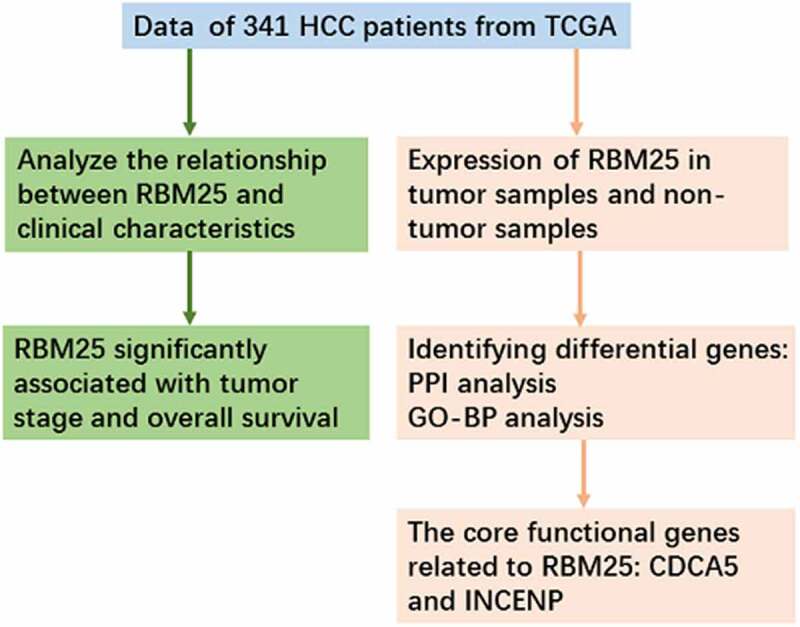

## Introduction

Hepatocellular carcinoma (HCC) is a common cancer worldwide and is a leading cause of cancer related death [1]. Although early stage HCC may be curable by resection, liver transplantation, or ablation, most patients present with unresectable disease and have a poor prognosis [2]. Although significant progress has been made concerning targeted drugs for HCC in recent years, overall survival (OS) remains poor [3]. For the multikinase inhibitors, sorafenib and lenvatinib, it has been found that sorafenib only modestly increases survival rates compared to placebo and lenvatinib is noninferior to sorafenib [4,5].The pathogenesis of HCC is extremely complex, involving cell cycle regulation, signal transduction, and other processes. This reflects the diverse functions and multiple interactions of many genes [6]. Understanding the pathogenesis and regulatory network of HCC is important to screen for therapeutic targets and to determine the mechanism of targeted drugs.

RNA alternative splicing is an important aspect of gene regulation. This splicing enables eukaryotic cells to produce more protein isoforms and thus to perform various biological functions [7,8]. RBM25 is a splicing factor that belongs to the RNA-binding protein family. It contains a common arginine-glutamate enriched core region that facilitates the localization of RBM25 around the splicing factor-enriched nucleosomes and its binding to RNA in the C-terminal PWI area [9]. RBM25 can interact with various splicing components, such as U small nuclear RNAs (snRNAs), to assemble spliceosome complexes and regulate splicing [10]. RBM25 can specifically regulate the selective splicing of Bcl-x pre-mRNA. Bcl-x is a member of the Bcl-2 family and can regulate cell apoptosis [11]. In addition, RBM25 is also involved in the regulation of multiple intracellular signal pathways, such as the AKT signaling pathway and the EPO pathway [12,13]. These findings indicate that RBM25 plays an important role in tissue and cell biology and participates in the regulation of cancer cell survival [14,15]. However, as the basic regulatory element of splicing, the regulatory mechanism of RBM25 in HCC remains unclear.

In the present study, we found that the expression of *RBM25* is significantly different between normal and tumor samples and significantly affects the survival of patients with HCC. We further explored the interaction of the RBM25 protein and the identification of related core functional genes to provide a basis for further studies of molecular regulatory pathways and splicing events.

## Materials and methods

### Datasets and acquisition of human tumor samples

The Cancer Genome Atlas (TCGA) database (https://portal.gdc.cancer.gov/) was searched for transcriptome data and corresponding clinical information of 373 HCC patient tissue samples and 50 normal samples.

### Bioinformatics analysis to identify RBM25 expression

The raw microarray data from the TCGA dataset were normalized using the *R affy* package. The normalized gene expression levels were expressed as log2-transformed values using a robust multi-array average. RBM25 gene expression was determined between the HCC tissue and normal tissue samples by the *R Limma* package. Significant differences in RBM25 expression between HCC and normal samples were determined using a threshold *P*-value <0.01 in the present study.

### Survival and clinicopathological characteristics analyses

Information of HCC patients obtained from the TCGA database was matched with the sample ID using the VLOOKUP index of EXCLE. After removing the missing data, the RBM25 expression values were ranked from top to bottom in the 341 HCC patients. Based on the median value of RBM25 expression, HCC patients were stratified into a high expression group and a low expression group. Furthermore, we explored the potential associations between RBM25 expression and survival rates and clinicopathological characteristics of HCC patients. In addition, correlations between the clinicopathological characteristics closely related to RBM25 expression and survival rate were analyzed in HCC. A *P*-value <0.05 was considered statistically significant.

### Differential expression analysis of TCGA data

The EdgeR *package* was used to perform a differential analysis of TCGA data by comparing the HCC and normal patient samples in the *R* programming environment. Genes with a differential expression of |log_2_ fold change (FC)| ≥ 1 at *P*< 0.05 were chosen as differentially expressed genes (DEGs) between the HCC and normal samples. Spearman coefficients between the DEGs and RBM25 were further calculated. DEGs with a *P*-value <0.05 were considered RBM25-related genes.

### Weighted gene co-expression network analysis (WGCNA)

The gene expression matrix of the TCGA samples was analyzed using the WGCNA algorithms of the *R* package. The expression of RBM25 was considered as a clinical trait in the co-expression network. All samples were clustered, and the outlier samples were excluded using the *flashClust* tool of *R*. The remainder were used for further analyses. To satisfy the distribution of a scale-free network, the *pickSoftThreshold* function was applied to calculate the soft-thresholding power. When the square of the correlation coefficient (R^2^) in the corresponding network reached 0.9, the optimal power value was determined to construct a scale-free topology network. Based on the soft threshold power value (power = 12) and mean connectivity, a gene clustering on topological overlap matrix (TOM)-based dissimilarity was obtained. The highly similar modules were combined with a dynamic tree-cutting algorithm, and the MEDissThres value was set to 0.25. These modules were related to the clinical traits. We focused on the hub module with the highest Pearson’s correlation coefficient. In addition, the hub module genes were overlapped with RBM25-related genes for subsequent analyses.

### Protein-protein interaction (PPI) network analysis

A PPI interaction network was constructed to explore the potential interaction among the identified genes. The overlapped RBM25-related genes were imported into the Search Retrieval of Interacting Genes (STRING) database (https://string-db.org/). The interactions of these genes provided by the STRING database were directly visualized using the *Cytoscape* software v3.7.2 (https://cytoscape.org/). The MCODE plug-in of the software was used for modularization analysis. The module with the highest score was designated Cluster 1.

### Enriched gene ontology-biological process (GO-BP) network analysis of RBM25-related genes

ClueGO was used to decipher functionally grouped GO and pathway annotation networks to understand their implication in BPs, molecular function, and cellular components. To systematically explore potential functions between the genes in Cluster 1, RBM25 and 39 RBM25-related genes were imported into *Cytoscape* software v3.7.2 (https://cytoscape.org/). Then, gene and pathway interaction networks were constructed using the ClueGO plug-in, based on Kappa statistics. In the network, each node represented a term, and the edge between two nodes indicated that the two BPs shared common genes. The color of the nodes reflected the enrichment and classification of the node (i.e., the functional group it belonged to).

### Immunohistochemistry (IHC) analysis of samples from the human protein atlas (HPA) database

The HPA (https://www.proteinatlas.org/) contains information for 26,000 human proteins and cell distribution information. Highly specific antibodies were used to examine the expression of each RBM25-related protein in 64 cell lines, 48 normal human tissues, and 20 tumor tissues by immunoassay (western blotting, immunofluorescence, and immunohistochemistry [IHC]) [16]. The expression of CDCA5 and INCENP proteins in normal and tumor tissues from the HPA database was analyzed by IHC staining. Staining intensity and quantity and patient information is available online.

### Statistical analyses

The *Wilcox* test was used to compare the expression levels of genes between normal samples and HCC tissue samples. The OS between the groups was compared using Kaplan-Meier (K-M) analysis with the log-rank test. Comparisons among the groups were performed using the *Kruskal-Wallis* test. A *P*-value <0.05 was considered statistically significant.

## Results

Alternative splicing of genes is a major post-transcriptional regulation mechanism that in many types of cancer. RBM25 is a poorly characterized RNA-binding protein and, as the basic regulatory element of splicing, its regulatory mechanism in HCC is still unclear. The integrated bioinformatics analysis associated higher expression of RBM25 with shorter OS in HCC patients. We further established that CDCA5 and INCENP are the core functional genes related to RBM25 in HCC.

### Expression of RBM25 in HCC

We initially evaluated the expression levels of RBM25 in multiple HCC patients from the TCGA database. There was a significant difference in RBM25 gene expression between tumor and normal samples. As delineated in Supplementary Figure 1a and 1b, the expression of RBM25 in tumor samples was significantly higher than that in normal samples **(*P*< 0.001)**.

### Associations between RBM25 expression and clinicopathological characteristics in HCC

As illustrated in Table 1, there were more males in both the RBM25 high and low expression groups **(60.52% vs. 75.29%, *P*= 0.016)**. There were obvious differences in pathological stage, N stage, and T stage between the two groups **(all *P*< 0.05)**. However, age and M stage of HCC patients were not significantly different in RBM25 expression **(both *P*> 0.05)**.

**Table 1.** Correlations between RBM25 expression with clinicopathologic features in 341 HCC patients

Characteristic		High(n = 171)	Low(n = 170)	P
Gender	female(n = 109)	67	42	0.016
	male(n = 232)	104	128	
Age, median(IQR),years	≤61(n = 184)	94	90	0.710
	>61(n = 157)	77	80	
Stage	Stage I(n = 168)	79	89	0.004
	Stage II(n = 83)	39	44	
	Stage III(n = 85)	51	34	
	Stage IV(n = 5)	2	3	
T	T1(n = 170)	81	89	0.006
	T2(n = 85)	41	44	
	T3(n = 76)	46	30	
N	N0(n = 249)	120	129	0.02
	N1(n = 4)	4	0	
	NX(n = 88)	47	41	
M	M0(n = 260)	124	136	0.081
	M1(n = 4)	1	3	
	MX(n = 77)	46	31	

### Associations between RBM25 expression and survival in HCC patients

**Figure 1.**
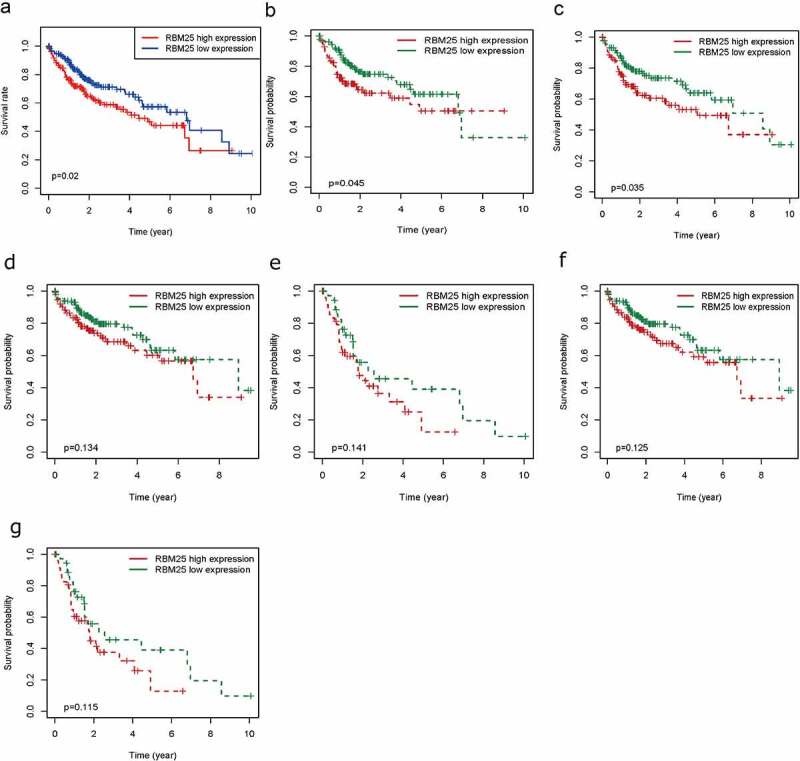
Elevated RBM25 predicated poor clinical outcome in HCC patients. Overall survival curve of high and low expression of RBM25 in (a) all HCC patients, (b) male patients, (c) N0 stage patients, (d) pathological stage I/II patients, (e) pathological stage III/IV patients, (f) T I/II stage patients, and (g) T III/IV stage patients

We investigated whether RBM25 expression had a significant effect on the survival of HCC patients. The results indicated that high expression of RBM25 in tumor tissues is considerably related to poor OS (Figure 1(a)**, *P*-value = 0.02)** in patients with HCC. Subgroup survival analyses in the different populations revealed an association of overexpression of RBM25 and poorer survival in males and N0 stage (Figure 1(b–c)**, *P*= 0.045 and 0.035, respectively)**. No significant differences were observed in pathological stage and T stage (Figure 1(d–g)**, both *P*> 0.05**), indicating that the expression of RBM25 may affect the pathogenesis of N0 stage in HCC.

### Identification of RBM25-related genes in HCC

**Figure 2.**
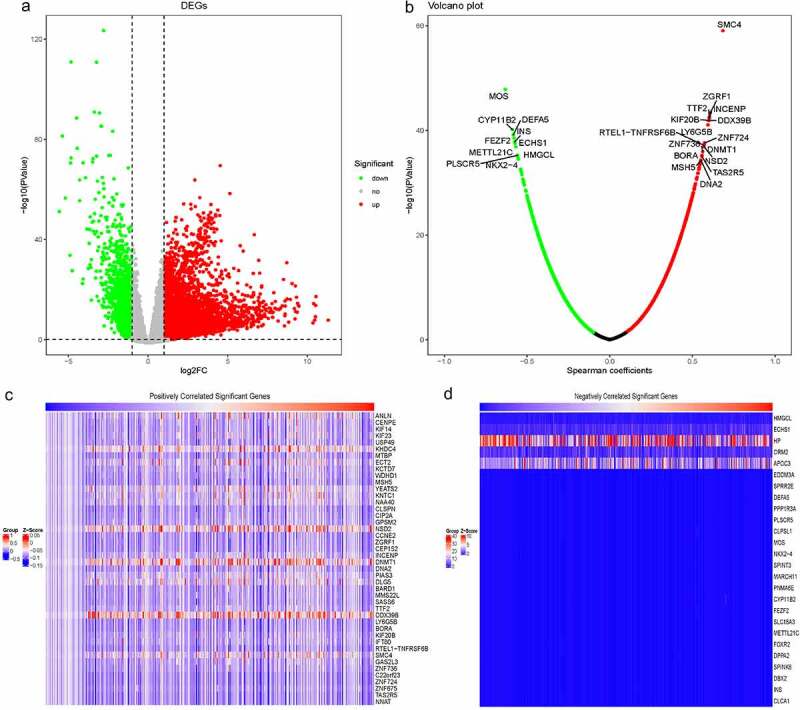
Identification of RBM25-related genes in HCC. (a) Comprehensive analysis of differential gene expression between normal tissues and tumor tissues. (b) RBM25-related genes with *P*< 0.05 were identified. A Volcano plot revealed the number of RBM25-related genes. (c and d) Z-scores of RBM25-positively-related genes (c) and -negatively-related genes (d) are displayed in a heatmap

To identify RBM25-related genes in HCC, we comprehensively analyzed the gene expression microarray data of the TCGA dataset. As displayed in Figures 2(a), 4861 DEGs were evident in HCC patients compared to the normal samples. Of these, 3775 were up-regulated and 1086 were down-regulated. Based on the Spearman coefficients between the DEGs and RBM25, RBM25-related genes with*P*< 0.05 were identified in HCC. A Volcano plot revealed the number of RBM25-related genes (Figure 2(b)). The Z-scores of RBM25-related genes with Spearman coefficients >0.5 were displayed in a heatmap (Figure 2(c–d)).

### WGCNA for RMB25-related genes

**Figure 3.**
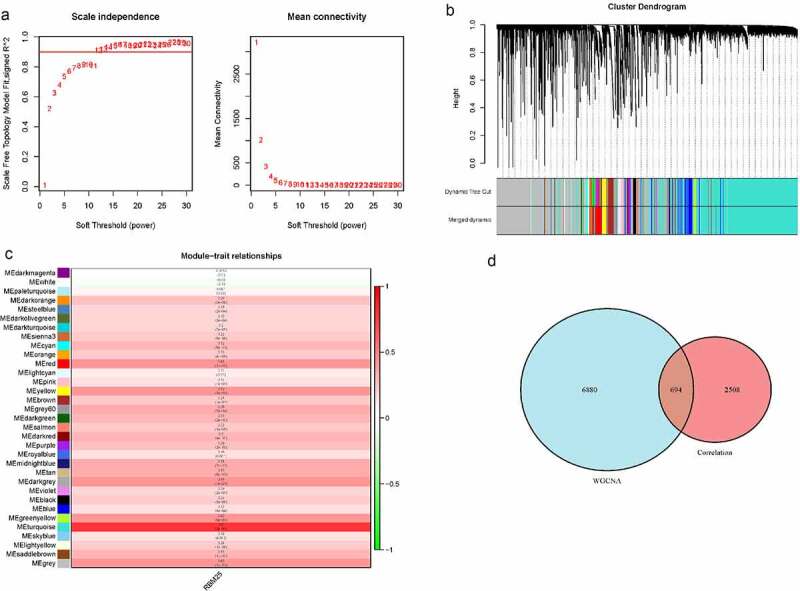
Weighted gene co-expression network analysis for RMB25-related genes. (a) The soft threshold power of β = 14 was considered to satisfy the distribution of a scale-free network. (b) A total of 33 modules with similar patterns were identified by merging similar modules. (c) The MEturquoise module had the highest Pearson coefficient with HCC (*Cor *= 0.8, *P*= 2e-91). (d) There were 694 genes from the MEturquoise module that were RBM25-related genes

To further identify the genes strongly related to RBM25 in HCC, we also performed WGCNA on the expression profile of the TCGA database. All samples were clustered, and no outlier was excluded in a hierarchical sample clustering (Supplementary Figure 2a). Supplementary Figure 2b depicts the clustering between normal samples and HCC patients after adding the clinical traits. The soft threshold power of β = 14 (scale-free R^2^ = 0.9) was the optional value to satisfy the distribution of a scale-free network (Figure 3(a)). After merging similar modules, a total of 33 modules with similar patterns were identified (Figure 3(b)). Correlation analyses between the traits and modules showed that the MEturquoise module had the highest Pearson coefficient with HCC. This was regarded as a key module for the selection of a gene signature (Figure 3(c), MEturquoise, ***Cor *= 0.8, *P*= 2e-91)**. Among a total of 7574 genes from the MEturquoise module, 3202 were RBM25-related genes. In addition, 694 common genes were obtained and further analyzed (Figure 3(d)).

### Construction of an RBM25-related gene and biological process network

**Figure 4.**
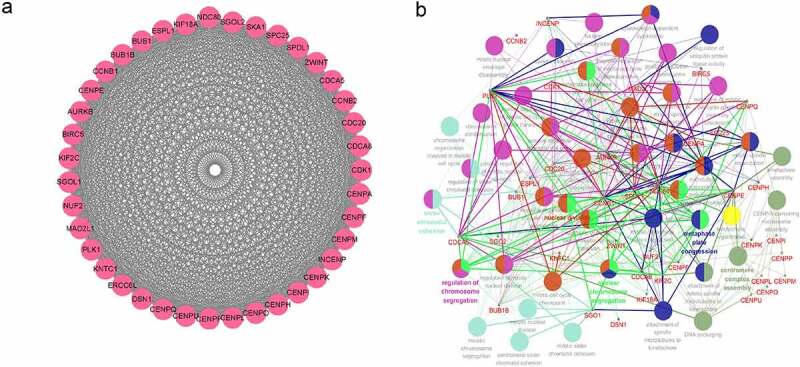
PPI and GO-BP analyses results. (a) PPI network analysis revealed 39 genes that were strongly positively correlated with RBM25. (b) GO-BP PPI interaction data for RBM25

To further explore the correlations between RBM25 and RBM25-related genes, a PPI network was constructed for modularization analysis. The 39 genes of Cluster 1 in the module with the highest score, and RBM25 are shown in Figure 4(a). The 39 genes were positively correlated with RBM25. To stringently investigate the biological functions of these RBM25 genes, a network between RBM25 and the 39 RBM25-related genes and GO-BP interaction was constructed using the ClueGO Plug-in in the Cytoscape software. As expected, BP terms that interacted with CDCA5 and INCENP were most prevalent, indicating the role of cross-talk in the network (Figure 4(b)). The results suggested that CDCA5 and INCENP might interact with RBM25 for regulating HCC progression.

### Expression patterns of the cross-talk genes CDCA5 and INCENP in HCC

To identify the genes that are significant for HCC progression, we acquired IHC pathological specimen data from HPA. Compared with the normal group, the expression levels of CDCA5 and INCENP were significantly increased in HCC patients (Supplementary Figure 3a**, all *P*< 0.01)**. Furthermore, the protein expression of CDCA5 and INCENP in HCC tissues was much higher than that in normal tissues (Supplementary Figure 3b**–C)**.

## Discussion

Emerging evidence has demonstrated that RBM25 is essential for several biological processes involved in regulating the proliferation and metastasis of tumor cells [11,12,14,15,17-19]. The present study is, to our knowledge, the first to demonstrate the relationship between the expression of RBM25 and HCC prognosis. We further established that CDCA5 and INCENP are the core functional genes related to RBM25. It may be important to further explore the molecular mechanisms and roles of these three genes in the occurrence and development of HCC.

Alternative splicing is a common method of gene regulation in eukaryotes. It is also important in the development of HCC [20,21]. While RBM25 has not been previously reported to be involved in HCC, it has been proven to be important in the occurrence and development of acute myeloid leukemia [15], prostate cancer [12,19], and colorectal cancer [22]. In acute myeloid leukemia, RBM25 controls the splicing of key genes. These include genes encoding the apoptotic regulator BCL-X and the MYC inhibitor BIN1 [15]. In addition, RBM25 binds directly to circAMOTL1L and induces its biogenesis whereas p53 regulates epithelial-to-mesenchymal transition via direct activation of RBM25 gene in human prostate cancer [12]. RBM25 can also specifically regulate the selective cleavage of Bcl-x pre-mRNA, thereby regulating cell apoptosis. This is closely related to tumor occurrence and development [15,23]. We found that the expression level of RBM25 in HCC patients was significantly higher than that in the normal group. Analyzing the relationship between RBM25 and the clinical characteristics of the HCC patients in the TCGA database revealed significant differences in the expression of RBM25 according to gender and the pathological stage. The survival was significantly different between the RBM25 high and low expression groups. These results suggest that the up-regulation of RBM25 expression promotes the occurrence and development of HCC.

To further explore the correlations between RBM25 and RBM25–related genes, a PPI network was constructed for modularization analysis. The results indicated that CDCA5 and INCENP might interact with RBM25 in regulating HCC. CDCA5 is a protein related to cell division. Any disorder in the process of cell division may lead to the formation of malignant tumors [24]. CDCA5 is the main regulator of sister chromatid separation and aggregation and has an important regulatory role in cell division [25,26]. Many recent studies have shown that CDCA5 can be used as a negative prognostic marker for HCC and have demonstrated that its regulatory cell division mechanism plays an important role in the occurrence and development of HCC [27-29]. A recent study suggested that RHPN1-AS1 can promote CDCA5 expression by functioning as a competing endogenous RNA for miR–485. Further, it can promote cell proliferation, metastasis, and apoptosis [28]. Another study showed that CDCA5, which is transcribed by E2F1, promotes oncogenesis by enhancing cell proliferation and inhibiting apoptosis via the AKT pathway in HCC [30]. In addition, INCENP dysregulation has also been confirmed to be related to the occurrence and development of a variety of tumors [31-34]. INCENP depletion can suppress neuroblastoma cell growth by inducing polyploidization, apoptosis, and senescence [31]. In addition, inherited variants in the INCENP of the chromosomal passenger complex contribute to the susceptibility of estrogen receptor negative breast cancer [35]. Further exploration of these three gene expression regulatory networks may provide new ideas for the study of molecular mechanisms of HCC.

In this study, we report the differential expression of RBM25 in HCC and analysis of the regulatory network of related genes. The expression of in HCC was significantly higher than that in normal liver tissues. This high RBM25 expression predicted poor prognosis in HCC patients. Unfortunately, we have not yet performed experiments exploring the potential carcinogenic mechanism of RBM25 in the development of HCC. It is not clear how RBM25 regulates CDCA5 and INCENP to affect the occurrence and development of HCC. Our detailed characterization of RBM25 protein interactions and related core functional genes provides a basis for further studies that will identify molecular regulatory pathways or splicing events that mediate the role of RBM25 in HCC occurrence and development.

## Supplementary Material

Supplemental Material

## Research highlights

- RBM25 was overexpressed in hepatocellular carcinoma (HCC) tissues
- RBM25 expression was associated with pathological stage and overall survival in HCC
- RBM25 might interact with CDCA5 and INCENP to regulate HCC progression
