# Supplementary material for: Prognostic alternative splicing regulatory network of RBM25 in hepatocellular carcinoma
Source: Bioengineered. 2021 Apr 8;12(1):1202–11. doi: 10.1080/21655979.2021.1908812 (PMC8806338; doi:10.1080/21655979.2021.1908812)
Supplement: Supplemental Material [file KBIE_A_1908812_SM2344.docx]

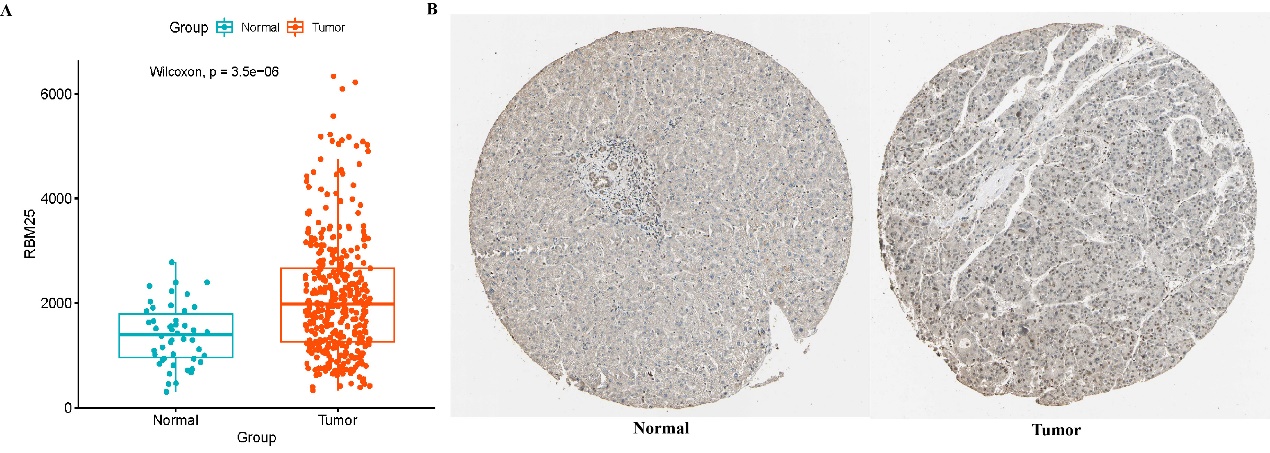


**Supplementary Figure 1:** RBM25 is overexpressed in HCC tissues. (A) The mRNA levels of RBM25 in tumor samples were significantly higher than those in normal samples (*P*<0.001). (B) IHC staining for RBM25 in normal tissues and tumor tissues.


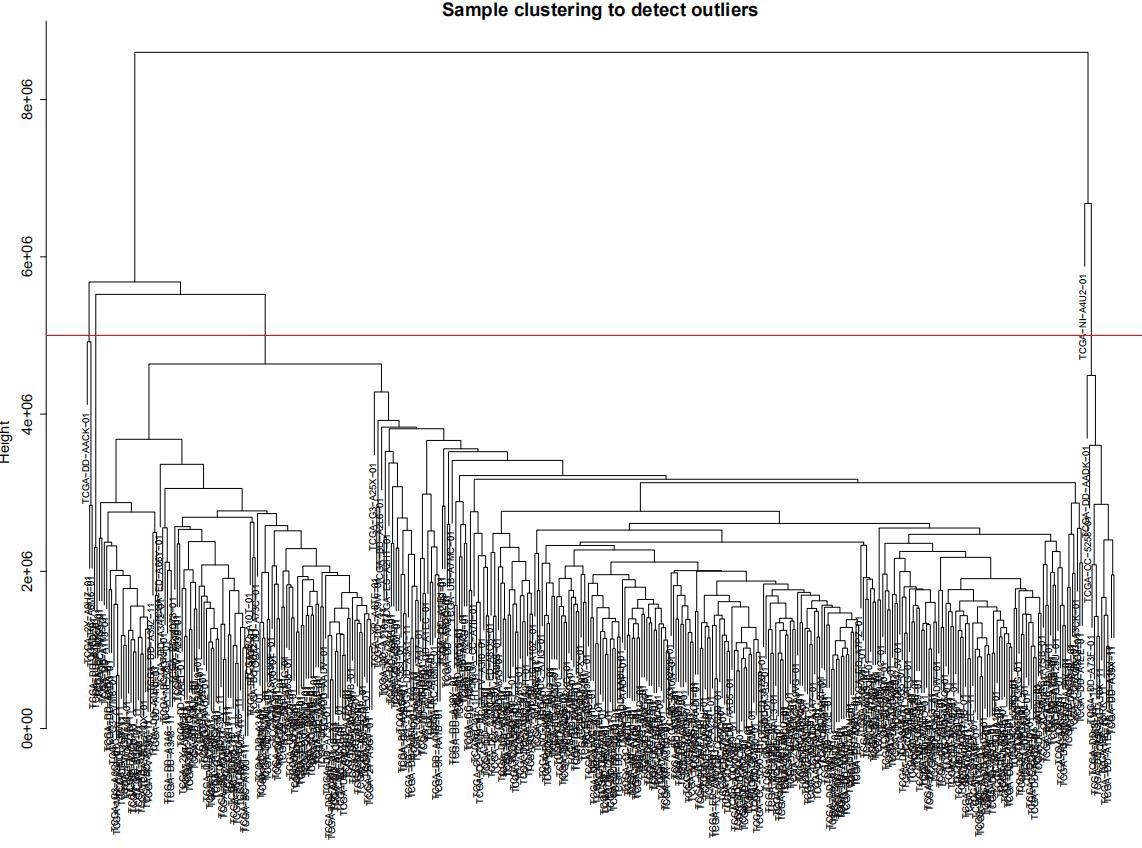


**Supplementary Figure 2A:** All samples clustering to detect outliers.


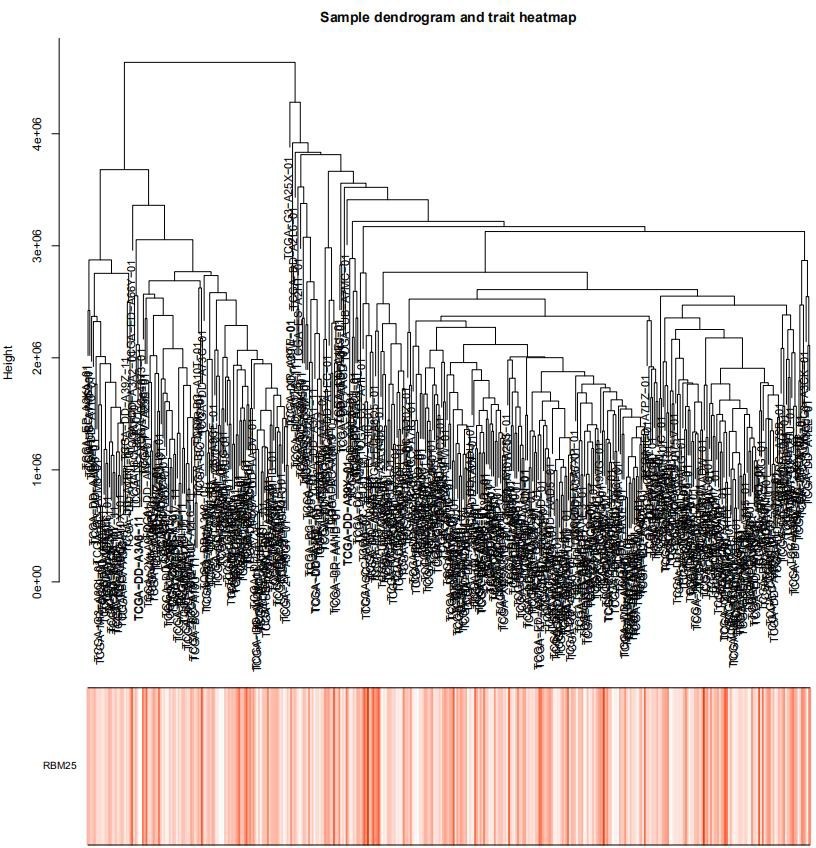


**Supplementary Figure 2B:** The sample dendrogram and trait heatmap.


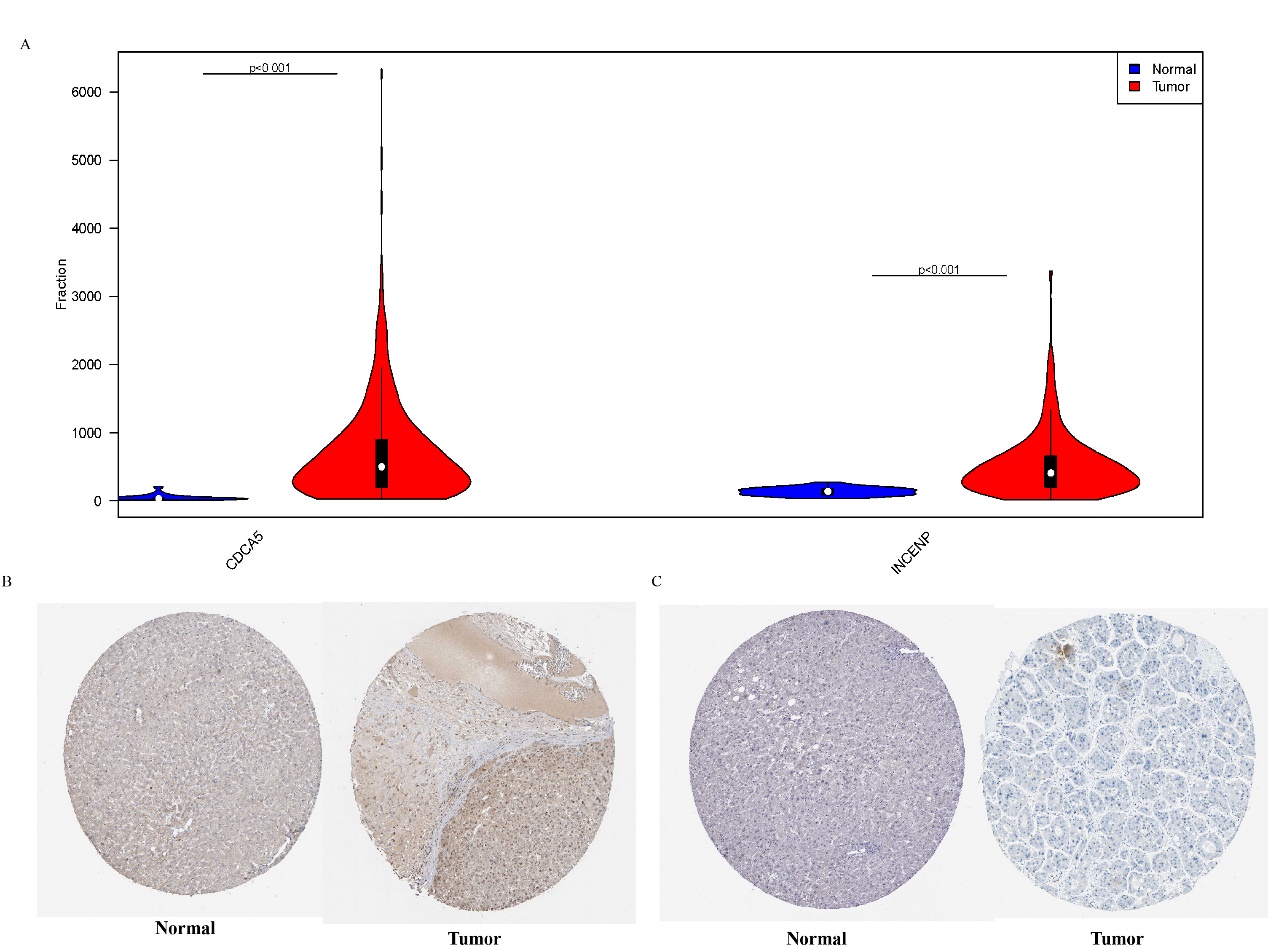


**Supplementary Figure 3:** Expression of cross-talk related genes CDCA5 and INCENP in HCC. (A) The mRNA levels of CDCA5 and INCENP in tumor samples were significantly higher than those in normal samples (*P*<0.001). (B and C) IHC staining for CDCA5 (B) and INCENP (C) in normal tissues and tumor tissues.
